# Reversible Evaporation and the Entropy of Black Holes

**DOI:** 10.3390/e28040455

**Published:** 2026-04-15

**Authors:** Friedrich Herrmann, Michael Pohlig

**Affiliations:** Faculty of Physics, Karlsruhe Institute of Technology, D-76128 Karlsruhe, Germany; pohlig@kit.edu

**Keywords:** black hole entropy, Hawking temperature, black hole evaporation, gravitothermal equilibrium

## Abstract

The entropy of a Schwarzschild black hole is commonly derived using thermodynamic relations whose physical interpretation is not always transparent, in particular with respect to the localization of temperature and entropy. In this paper, we present a derivation of the Bekenstein–Hawking entropy based exclusively on the principles of phenomenological thermodynamics, formulated entirely in regions where spacetime is effectively flat. The analysis considers a reversible evaporation process in which the black hole is surrounded by a tunable thermal radiation bath whose temperature is kept arbitrarily close to the Hawking temperature. In this limit, entropy production can be made negligible. By integrating the entropy flux through a distant reference surface over the evaporation process, the standard entropy formula is obtained without invoking assumptions about the localization of the black hole entropy or about microscopic degrees of freedom. The derivation is mathematically simple but conceptually instructive. The approach is intended to be accessible to readers familiar with classical thermodynamics and general relativity at an advanced undergraduate or graduate level.

## 1. Introduction

In his seminal 1975 paper [1], Hawking demonstrated that a black hole behaves as a black body, i.e., it emits thermal electromagnetic radiation. This result came as a surprise even to Hawking himself, since it had long been assumed that nothing could escape from the area below the Schwarzschild horizon.

Hawking calculated the temperature $T_{H}$ of this radiation as measured far away from the black hole [2], where spacetime is effectively flat and the gravitational field no longer significantly alters the radiation spectrum:


(1) $$T_{H}=\frac{\hbar c^{3}}{8\pi k_{B}G}\frac{1}{M}$$

Here, ℏ, c, $k_{B}$, and G denote fundamental constants, and M is the mass of the black hole.

This discovery provided the decisive impetus for the development of black hole thermodynamics. One of its central questions concerns the entropy of black holes. Hawking initially rejected the idea that black holes could possess entropy at all [3]. In fact, an explicit expression for black hole entropy had been proposed by Bekenstein [4], even before Hawking’s calculation of the radiation temperature, and was later confirmed by Hawking himself [5]. Bekenstein’s original result differed by a factor of four; the expression now generally accepted is

(2) $$S=4\pi \frac{k_{B}G}{\hbar c}M^{2}$$

Because of the appearance of several fundamental constants, Equations (1) and (2) look rather complicated. Their physical content, however, is simple: the Hawking temperature is inversely proportional to the black hole mass, whereas the entropy is proportional to the square of the mass.

The existing literature (see, for example, Wald [6], Wallace [7] or Jacobson et al. [8]) often gives the impression that deriving the entropy of a black hole is a highly nontrivial problem that cannot be addressed using classical thermodynamics alone.

In addition, in our view, some derivations presented in the literature are not always conceptually transparent. To illustrate this, let us briefly recall a standard derivation, for example, that presented by Susskind [2].

One assumes that the thermodynamic relation

(3) dE=TdS

applies to a black hole, where E is the energy, T is the absolute temperature, and S the entropy.

Writing $dE=c^{2}dM$ and substituting the Hawking temperature $T_{H}$ for T, one obtains

(4) $$dS=\frac{c^{2}dM}{T}=\frac{8\pi k_{B}G}{\hbar c}MdM$$

If one now imagines the black hole evaporating completely, so that its mass decreases from an initial value M to zero, integration over M yields Equation (2).

The difficulty with this derivation lies in the interpretation of Equation (3). In classical thermodynamics, the differentials dE and dS refer to changes in extensive quantities within a spatial region that is assumed to be in internal thermal equilibrium, i.e., to have a well-defined and homogeneous temperature. It is therefore unclear to which spatial region Equation (3) is meant to apply in the context of black holes. In particular, which region is supposed to have the temperature $T_{H}$?

This conceptual issue is often sidestepped by statements such as that the surface gravity of a black hole is “analogous to temperature” [1,9], that a black hole is “endowed” with a temperature [10], or that it is “characterized” by a temperature and an entropy [11]. Elsewhere, it is asserted that the surface gravity (divided by 2π, if $c=k_{B}=\hbar =1$) is the actual physical temperature of a black hole ([6], p. 12). Taken together, these interpretations weaken the persuasiveness of the arguments.

In this paper, we show how the entropy Formula (2) can be derived directly from Hawking’s expression (1) for the radiation temperature using only the tools of phenomenological thermodynamics. In doing so, we avoid the conceptual ambiguities mentioned above. In particular, we do not need to make any assumptions about the microscopic nature of black hole entropy, its spatial distribution, or the mechanism of entropy production.

In our approach, we consider the “evaporation” of a black hole in a reversible process. No new entropy is generated while the Hawking radiation is emitted. To this end, we imagine the black hole to be surrounded, at a large distance, by a spherical heat reservoir whose temperature can be freely adjusted.

The key thermodynamic quantities in our derivation—energy flow, entropy flow, and temperature—are then defined at locations far outside the black hole, where spacetime is effectively flat and classical thermodynamics can be applied without ambiguity.

Before presenting the actual calculation, which is short and mathematically identical to the standard derivations, we make a few preliminary remarks. In Section 2, we illustrate the magnitude of the effects involved. In Section 3, we describe the thought experiment that allows us to use a simple and robust thermodynamic relation to calculate the entropy. Finally, in Section 4, we derive Equation (2) in just a few lines.

## 2. What We Are Talking About

To gain some intuition for Equation (2), let us evaluate the temperature and entropy of a typical stellar-mass black hole with a mass of three solar masses. We take

$$M=6\cdot 10^{30}\;kg.$$

Using Equation (1), the Hawking temperature of the emitted electromagnetic radiation is

$$T_{H}=2.1\cdot 10^{-8}\;K.$$

From Equation (2), the entropy of the black hole is

$$S=1.8\cdot 10^{32}\;J/K.$$

The temperature is extremely low. To appreciate the enormous magnitude of black hole entropy, it is useful to compare it with more familiar systems.

Consider first a thought experiment in which a block of iron of mass 1 kg is dropped into the black hole. We compare the entropy $S_{block}$ of our block before falling in at room temperature (approximately 300 K) with the increase $\Delta S_{bh}$ of the black hole entropy due to the infall.

The entropy of the iron block is roughly

$$S_{block}=500\;J/K.$$

Using Equation (2), the increase in black hole entropy becomes

$$\Delta S_{bh}=4.4\cdot 10^{24}\;J/K.$$

We see that the entropy of the black hole has increased dramatically. The entropy content of the body before falling in is completely negligible compared to the increase in entropy during the fall.

As a second comparison, imagine dropping the same block into a one-meter-deep hole in the ground on Earth. The entropy produced by the inelastic impact at the bottom of the hole is approximately 0.03 J/K, which is very small compared to the pre-existing entropy of the block.

In order to produce an amount of entropy when falling into the hole that is comparable to the block’s initial entropy, the hole in the ground would have to be about 30 km deep. This value gives us an impression of how deep the hole would have to be in order to generate as much entropy upon impact of the block as when falling into a singularity.

Finally, consider the entropy budget of the observable universe. While stars, starlight, the cosmic microwave background, the cosmic neutrino background (relic neutrinos), dark matter, and the gravitational wave background (relic gravitons) together contribute roughly $10^{67}\;J/K$, the entropy of stellar-mass black holes is already of order $10^{74}\;J/K$. Supermassive black holes contribute another seven orders of magnitude more [12,13].

## 3. Tools

Our derivation relies on two ideas:

- We consider only quantities defined at points or surfaces in effectively flat spacetime.
- We only consider states of the radiation field that are close to equilibrium states.

We imagine a spherical reference surface A surrounding the black hole at a sufficiently large radius that gravitational effects are negligible. At this surface, the temperature of the outgoing radiation is the Hawking temperature given by Equation (1).

We are interested in the energy flux P and the entropy flux $I_{S}$ crossing the surface A. We consider a process in which the black hole evaporates completely and integrate these fluxes over time.

Energy conservation implies that the total energy passing through A equals the initial energy of the black hole. This is not automatically true for entropy, since entropy is produced when blackbody radiation is emitted into empty space: the emitter and its surroundings are not in thermal equilibrium.

To avoid this complication, we modify the thought experiment. Instead of placing the black hole in a radiation-free vacuum, we enclose it in a cavity filled with thermal radiation [7]. The temperature of this heat bath is adjustable and is always chosen to be slightly below the Hawking temperature. As the black hole evaporates and $T_{H}$ increases, the bath temperature is adjusted accordingly. Throughout the process, we require

(5) $$\Delta T\ll T_{H}$$

where ΔT is the temperature difference between the Hawking radiation and the surrounding heat bath.

This construction has two important consequences. First, the relation between energy flux and entropy flux reduces to the familiar equilibrium form

(6) $$P=T\cdot I_{S}$$

(For radiation into an initially radiation-free vacuum, a factor of 3/4 would appear on the right-hand side of Equation (6) [14]).

Second, the entropy production during emission can be made arbitrarily small compared to the entropy transferred. According to the second law, the entropy balance equation

$$\partial_{\mu }s^{\mu }=\sigma$$

contains a production term σ≥0 [15]. Here, $s^{\mu }$ is the entropy density four-vector. In our setup, σ can be made arbitrarily small, so that

$$\partial_{\mu }s^{\mu }\approx 0$$

The system is therefore in a state of local equilibrium. This statement applies to the entire system consisting of the black hole and the radiation field inside the reference surface, even though the spacetime distribution of entropy inside the horizon is unknown.

We can now proceed to calculate the entropy of the black hole by integrating Equation (4).

The reader may still have a problem with the statement that our system, consisting of the black hole and the radiation field below the reference surface A, is in a state of local equilibrium. We discuss this problem in the Appendix A.

## 4. Calculation of the Entropy Content of the Black Hole

The relationship between P and $I_{S}$ is given by Equation (6), where we substitute $T_{H}$ for the temperature:

$$P=T_{H}\cdot I_{S}$$

Thus,

(7) $$I_{S}=\frac{1}{T_{H}}P$$

During evaporation, the entire entropy of the black hole flows through the surface A. Integrating the entropy flux over time from the initial time $t_{1}$ (mass M) to the final time $t_{2}$ (mass zero), we obtain

$$\Delta S=\int_{t_{1}}^{t_{2}}I_{S}dt$$

The entropy of the black hole in its initial state is equal to the total entropy emitted:

$$S_{bh}=-\Delta S$$

Using Equation (7), this becomes

$$S_{bh}=-\Delta S=-\int_{t_{1}}^{t_{2}}\frac{1}{T_{H}}Pdt$$

With (1) and

$$Pdt=\frac{dE}{dt}dt=c^{2}dm$$

(E = energy, m = mass).

We finally obtain

$$S_{bh}=-\int_{t_{1}}^{t_{2}}\frac{1}{T_{H}}Pdt=-\frac{8\pi k_{B}G}{\hbar c}\int_{M}^{0}mdm=4\pi \frac{k_{B}G}{\hbar c}M^{2}$$

We have thus obtained the well-known expression for the entropy of the black hole without referring to processes that take place in the vicinity of the black hole or below the event horizon. Our only assumption was that the entire system, consisting of the black hole and its surroundings, is in equilibrium.

## 5. Conclusions

For readers familiar with classical thermodynamics, the usual derivations of black hole entropy may appear unnecessarily opaque or even conceptually questionable. As we have shown, the entropy formula can be obtained with minimal effort using the simplest tools of phenomenological thermodynamics, provided two ideas are adopted:

- One restricts attention to quantities defined far away from the black hole, where spacetime is effectively flat.
- The emission process is realized reversibly.

It is not surprising that the final expression coincides with the standard result. The novelty lies not in the mathematics, but in the interpretation of the thermodynamic relations involved.

## Data Availability

Data are contained within the article.

## Appendix A. Gravitothermal Equilibrium and Local Temperature

The temperature measured by a stationary observer (a “fiducial” observer [2]) outside a Schwarzschild black hole increases as the horizon is approached and diverges at the horizon. It is given by

$$T(r)=\frac{T_{H}}{\sqrt{1-\frac{r_{S}}{r}}}$$

Here, r is the radial coordinate of the Schwarzschild metric (i.e., the circumference of a circle with the singularity at its center divided by 2π), and $r_{S}$ is the Schwarzschild radius.

At first sight, this strong temperature gradient seems to imply a substantial outward entropy flow accompanied by enormous entropy production [16]. This conclusion, however, is incorrect because it neglects the coupling between energy and entropy transport in a gravitational field.

If the entropy of electromagnetic radiation is subjected to a “drive”, i.e., a temperature gradient, this also causes an energy transport, and if the energy (=mass) is subjected to a drive, i.e., a gravitational potential gradient or a corresponding space–time curvature, the entropy is also affected. A combination of the two drives is responsible for the coupled energy–entropy transport [17]. We call the “combined potential” responsible for the “net drive” the gravitothermal potential:

$$\Theta (r)=\sqrt{g_{00}}\cdot T(r)$$

where

$$g_{00}=1-\frac{r_{S}}{r}$$

is the time–time component of the metric tensor of the Schwarzschild metric.

Thus, we get

$$\Theta (r)=T_{H}$$

which is independent of the radial coordinate.

Thus, the net driving force for the coupled energy–entropy transport vanishes everywhere. The system is in a state that may appropriately be called gravitothermal equilibrium. The relation between energy flux P and entropy flux $I_{S}$ takes the form

$$P=\Theta (r)\cdot I_{S}$$

and is valid for any spherical surface outside the horizon, not only in the asymptotically flat region.

This situation is closely analogous to electrochemical equilibrium, where electrical and chemical potentials combine to yield a spatially constant electro-chemical potential, even across a p–n junction. Similar concepts appear in meteorology in the form of potential temperature [18], or in relativistic thermodynamics as thermokinetic potentials governing entropy transport between moving bodies [19].

The quantity Θ is a local scalar whose value is equal to the Hawking temperature. Apparently, there was a tendency to assign a local interpretation to this quantity at the horizon, where it became known as surface gravity. Indeed, the Hawking temperature is proportional to the surface gravity κ:

$$T_{H}=\frac{\hbar }{2\pi k_{B}c}\kappa$$

with

$$\kappa =\frac{c^{4}}{4GM}$$

Using the Schwarzschild radius

$$r_{S}=\frac{2GM}{c^{2}}$$

this can be rewritten as

$$\kappa =\frac{GM}{r_{S}^{2}}$$

which formally resembles the Newtonian gravitational field strength at the horizon. It should be emphasized, however, that this quantity cannot be directly measured at the horizon.
